# Why does the metabolic cost of walking increase on compliant substrates?

**DOI:** 10.1098/rsif.2022.0483

**Published:** 2022-11-30

**Authors:** Barbara Grant, James Charles, Brendan Geraghty, James Gardiner, Kristiaan D'Août, Peter L. Falkingham, Karl T. Bates

**Affiliations:** ^1^ Department of Musculoskeletal & Ageing Science, Institute of Life Course & Medical Sciences, University of Liverpool, The William Henry Duncan Building, 6 West Derby Street, Liverpool L7 8TX, UK; ^2^ School of Biological and Environmental Sciences, Liverpool John Moores University, James Parsons Building, Bryon Street, Liverpool L3 3AF, UK

**Keywords:** biomechanics, locomotion, energetics, musculoskeletal model, compliant substrate

## Abstract

Walking on compliant substrates requires more energy than walking on hard substrates but the biomechanical factors that contribute to this increase are debated. Previous studies suggest various causative mechanical factors, including disruption to pendular energy recovery, increased muscle work, decreased muscle efficiency and increased gait variability. We test each of these hypotheses simultaneously by collecting a large kinematic and kinetic dataset of human walking on foams of differing thickness. This allowed us to systematically characterize changes in gait with substrate compliance, and, by combining data with mechanical substrate testing, drive the very first subject-specific computer simulations of human locomotion on compliant substrates to estimate the internal kinetic demands on the musculoskeletal system. Negative changes to pendular energy exchange or ankle mechanics are not supported by our analyses. Instead we find that the mechanistic causes of increased energetic costs on compliant substrates are more complex than captured by any single previous hypothesis. We present a model in which elevated activity and mechanical work by muscles crossing the hip and knee are required to support the changes in joint (greater excursion and maximum flexion) and spatio-temporal kinematics (longer stride lengths, stride times and stance times, and duty factors) on compliant substrates.

## 1. Introduction

The evolution of animal locomotion has mostly occurred on substrates with complex heterogeneous topography and material properties. However, our current understanding of animal gait and energetics is dominated by studies on hard, level surfaces in laboratories, which do not reflect most naturally occurring terrains. Recent work on humans has shown that locomotion on complex substrates like loose rock surfaces [1], ballast [2], uneven [3,4] and compliant [5–10] terrains is typically associated with an increase in energy expenditure relative to uniform, non-deforming substrates. Indeed, variations in the compliance or stiffness of footwear has also been shown to systematically affect locomotor costs [11,12]. The term ‘compliant’ has been used broadly within the field [4–9] to refer to any substrate that has non-negligible deformation under loads typically generated during human locomotion. A substantial body of literature has sought to understand elevated energetic costs on compliant substrates like sand, mud and snow [5–7,13], but at present there remains little consensus about the primary mechanistic causes.

Lejeune *et al*. [7] and Zamparo *et al*. [6] compared the change in the energetic cost of transport (CoT) on sand across a range of speeds. These studies discovered different magnitudes and nature of change in CoT with speed on compliant sands and invoked different biomechanical mechanisms to explain these increases. Lejeune *et al*. [7] attributed the higher energetic costs to an increase in muscle-tendon work and a decrease in muscle-tendon efficiency whereas Zamparo *et al*. [6] proposed that it was due to a lower energy recovery through a reduction in the efficiency of pendular energy exchange in walking and in the reduced recovery of elastic energy storage in running.

Pinnington & Dawson [8] suggested a potential increase in muscle co-activation and an increase in foot contact time on compliant substrates may lead to increased oxygen consumption due to a reduction in elastic energy storage and recovery, and ultimately a decrease in muscle-tendon efficiency. These authors noted that foot slippage may also play a role, as postulated by Zamparo *et al*. [6]. Voloshina *et al*. [3] found an increase in mean muscle activity and increased mechanical work on uneven substrates and suggested there may be a potential increase in muscle co-activation. Bates *et al*. [14] speculated that increased activation of ankle extensors, specifically, may be a major contributor to increased CoT on sand. Pandolf *et al*. [13] proposed that increasing work to lift the centre of mass (CoM), a stooping posture and difficulties maintaining stability are the primary causes of increased CoT when walking on snow.

Therefore, while it is widely accepted that compliant substrates incur an increase in CoT, there remains considerable uncertainty about the relative contribution of different biomechanical factors underpinning this increase. Possible reasons include the measurement of different variables across studies [10], variation in footwear (e.g. barefoot, different types of shoes; but see [8]), substrates used, and the gaits and speeds tested. Unfortunately, the absence of quantification of the mechanical properties of the compliant substrates used across past studies impedes comparison. In this study, we attempt to address these issues and provide an exhaustive evaluation of why the energetic cost of walking increases as substrate compliance increases. To achieve this, we present a large experimental kinematic and kinetic dataset of human walking on foams of differing thickness, with detailed characterization of substrate mechanical properties by uniaxial compression testing. Quantification of substrate properties not only facilitates repeatability and systematic comparison with other substrates but also allows us to carry out subject-specific computer simulations of locomotion across compliant substrates. This validated individualized computational framework [15] allows for the prediction of aspects of internal kinetics and muscle performance that cannot be measured non-invasively and thus may provide further insights into the mechanisms behind locomotor cost beyond those allowed by experimental methods alone. Through this integrated experimental-computational workflow we test the previously proposed hypotheses that increased CoT on compliant substrates is primarily the result of (HYP1) negative disruption to pendular energetic exchange [6], (HYP2a) increased muscle activation throughout the support limb [3] or (HYP2b) within specific muscle groups [14], (HYP3) increased musculotendon unit (MTU) work and decreased efficiency [7] and/or (HYP4) correcting greater instabilities indicated by increased variability in gait [13].

## 2. Material and methods

### 2.1. Experimental data collection

A total of 30 young, healthy individuals (15 males, 15 females; age = 27.4 ± 3.8 years; height = 1.76 ± 0.1 m; body mass = 71.1 ± 9.0 kg; body mass index = 23.0 ± 2.1 kg m^−2^; see electronic supplementary material, table S1; Exclusion Criteria Text, S1) signed informed consent before participating in the study in accordance with ethical approval from the University of Liverpool's Central University Research Ethics Committee for Physical Interventions (no. 3757). Data were collected as part of a larger study [16]. As described in this previous study, we used a K5 wearable metabolic unit (COSMED, Rome) to measure and quantify the energy efficiency of walking of each subject on different types of terrain. Oxygen uptake (VO_2_ ml O_2_ s^−1^) and carbon dioxide produced (CO_2_ ml O_2_ s^−1^) were measured continuously during 7 min of barefoot walking in a breath-by-breath analysis on three surfaces: (i) hard, level floor; (ii) a 13.2 m long compliant polyether polyurethane foam with a thickness of 6 cm (thin foam); and (iii) the same foam of 13 cm thickness (thick foam) (eFoam.co.uk. Medium foam. Density range: 31–34 kg m^−3^, Hardness strength: 100–130 Nm; see electronic supplementary material, figure S1). Subjects walked back and forth across the walkways continuously at a self-selected speed during the 7 min periods. From these data, Charles *et al*. [16] previously found that walking cost of transport (CoT) significantly increased with foam thickness (*p* ≤ 0.05; electronic supplementary material, figure S2), with CoT highest on the thick foam (14.25 ± 3.17 ml O_2_ m^−1^), and lowest on the floor (8.02 ± 1.84 ml O_2_ m^−1^) (electronic supplementary material, figure S2).

Not discussed or analysed in this previous study [16], all participants also had three-dimensional kinematics, ground reaction forces and surface electromyography (EMG) measured synchronously during trials. During the continuous walking on each substrate, the foams were placed over three in-series force plates (Kistler 9281E) in the centre of their length, with three-dimensional kinematics, ground reaction forces (GRFs) and EMG recorded for 30 s at every minute from 3 min onwards. To increase sample size and examine gait changes outside the context of longer, continuous bouts of walking, an additional 15 single trials were collected where a participant completed a single continuous passage across the substrates (with substrate order randomized) while only three-dimensional kinematics and EMG were measured. For all trials, whole-body kinematics were recorded at 200 Hz using 69 reflective markers and a 12-camera Qualisys Oqus 7 motion capture system (Qualisys Inc., Götenborg, Sweden). Kinematic data processing was undertaken in Visual3D (C-Motion Inc., Germantown, MD, USA) with a kinematic model comprising 13 segments: bilateral feet, shanks, thighs, upper arms, forearms and head, trunk and pelvis. From this data, Visual3D calculated CoM motions by using the position of the kinematic model in relation to the laboratory based on mechanical principle patterns [17]. Gait events were calculated automatically using a coordinate-based algorithm [18] but checked manually for every trial. Heel-strike was taken as the first weight-bearing contact between the substrate and the foot, and toe-off was taken as the last weight-bearing contact between the substrate and the hallux.

Marker tracking and EMG registration were all synchronized. EMGs were recorded using the wireless Trigno EMG (Delsys, MA, USA) system at a sampling rate of 1110 Hz. Standard EMG skin preparation methods were used [19] and the electrodes were positioned to record the activity of eight left lower extremity muscles: biceps femoris (BFL), rectus femoris (RF), vastus lateralis (VL), vastus medialis (VM), tibialis anterior (TA), lateral gastrocnemius (LG), medial gastrocnemius (MG) and soleus (SOL). Due to synchronization issues, EMG data for participants 1–6 were not included. All EMG processing was performed in Matlab v. 2019b (Mathworks, Natick, USA). The raw EMG signals were high pass filtered at 12 Hz with a second-order Butterworth filter, full-wave rectified and cropped to individual gait cycles. These data were then normalized (nEMG) to maximum amplitude during all walking trials to allow for between-participant comparison, and the integrated values were calculated (iEMG).

Mechanical energy data was processed in MATLAB and yielded gravitational potential energy (*E*_pot_), kinetic energy (*E*_kin_) and total mechanical energy (*E*_tot_) of the mass-normalized three-dimensional CoM. The recovery of mechanical energy (expressed as a percentage; R), relative amplitude (RA) and congruity (the time when potential energy and kinetic energy are moving in the same direction; CO) were calculated [20].

### 2.2. Statistical analysis of experimental data

Joint kinematics were analysed using two statistical approaches: one-dimensional statistical parametric mapping (1D-SPM) [21], and linear mixed-effect models (LMMs). 1D-SPM has the benefit of allowing continuous statistical analysis without treating time points as independent but does not allow incorporation of additional factors (e.g. random or fixed effects) as LMMs do. 1D-SPM analyses were performed using Matlab to compare hip, knee and ankle joint angles across substrates, with null hypothesis of no difference and alpha of 0.05. Joint angles at gait events (heel-strike and toe-off), spatio-temporal data, iEMG data and mass-normalized mechanical energy exchange variables were analysed using LMMs, where restricted maximum likelihood was used to assess the significance of the fixed effects, substrate and trial type (continuous walking and single trials) in explaining variation. As gait speed [22] and gender [23] can have an effect on gait biomechanics, LMMs were repeated with the inclusion of speed and gender set as fixed effects. Subjects were set as random effects, which allowed different intercepts for each subject. All LMMs were performed in R [24] using the lmer function in the R package lme4 [25] and lmerTest [26]. The coefficient of variation (CV) was calculated for all spatio-temporal data as a measure of gait variability. Examples of the R and Matlab code used above are provided in the electronic supplementary material.

### 2.3. Material testing of substrates

Mechanical behaviour of the thin and thick foam substrates was characterized by uniaxial compression using an Instron 3366 universal testing machine (UTS) with a 2350 series 5 kN load cell (Instron, Norwood, MA) attached. A 203 mm diameter flat indenter foot was connected to the load cell by means of a swivel joint and the UTS was fitted with a bespoke horizontal base plate to support the samples during testing. The base plate was perforated with 6.5 mm diameter holes at 20 mm centres to allow for rapid escape of air from the sample during the test [27]. Initial trials were carried out to assess the effect of cyclic loading and strain rate on the samples. Ultimately, one 380 × 380 mm sample of each thickness was subjected to a single loading cycle at a rate of 500 mm min^−1^ up to a compressive strain of 90%. The indenter load and displacement were recorded and used to calculate the corresponding compressive strain, stress and modulus of the foam substrates. Collectively, these data were used to provide gross quantification of the mechanical behaviour of the foams for repeatability and comparability to other substrates, and to derive simplified representations of material properties required for multi-body dynamics (MDA) analysis (for further detail, see electronic supplementary material, text S2–S3).

### 2.4. Multi-body dynamics analysis

**Figure 1.**
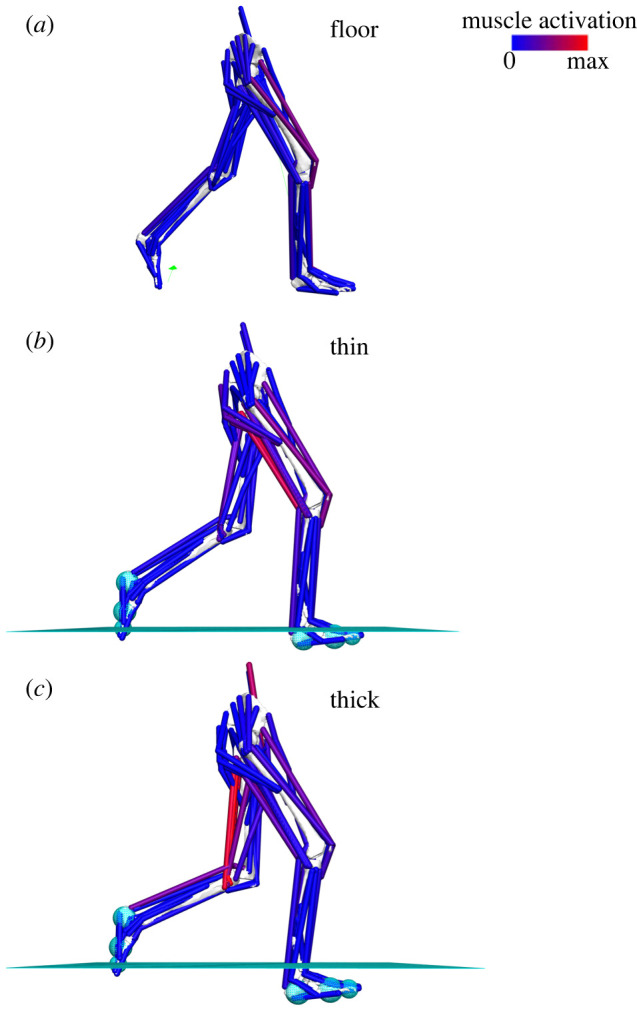
Lateral views of the subject-specific models and simulations of walking on the (*a*) floor, (*b*) thin foam and (*c*) thick foam, with predicted muscle activations shown. The cyan planes in (*b*) and (*c*) represent the top surface of the foams.

To investigate potential internal kinetic mechanisms behind differences in CoT between the hard floor and foam surfaces, one walking cycle was simulated over each substrate with one subject-specific, 12 joint degree of freedom, 92 musculotendon unit (MTU) actuated lower limb musculoskeletal model in OpenSim 4.2 [28] (figure 1; age = 23, height = 180 cm, body mass = 77.4 kg; BMI = 23.8 kg m^−2^). This model is part of a previously published set of subject-specific models [15] and freely available at the following link (doi:10.17638/datacat.liverpool.ac.uk/1536). Note that in the previous study and its data deposit [15], the model is referred to as Subject 4. In this current study, the participant is labelled as Subject 9 (electronic supplementary material, table S1). This model included muscle-force generating properties from the subject's MRI that was matched to the subject's own kinematics collected in this study. This subject was selected as their lower limb kinematics during walking on all substrates fell entirely within one standard deviation of the means for all subjects throughout each gait cycle (electronic supplementary material, figure S3). Inverse kinematics was used to generate the generalized coordinates of each unlocked degree of freedom from the motion capture marker positions, and computed muscle control (CMC) was used to predict muscle activations and powers during walking over each surface. Experimentally measured GRFs recorded during the floor walking trials were applied to the model to simulate walking on the hard floor. Contact geometries were used to simulate contact between the foot and the foam surfaces during the thin and thick foam walking simulations. Here, contact spheres were placed at the CoM of the calcaneus, forefoot and toes bodies of each lower limb to represent the soft tissue of each foot segment, while a contact half-space was placed at different heights to represent each foam surface (thin foam = 6 cm; thick foam = 13 cm). In OpenSim, the contact forces between each sphere and the foam surfaces were defined as Hunt–Crossley forces [29], where the stiffness parameters were set at 0.047 MPa (47 005 N m^−2^) for the thin foam and 0.029 MPa (28 763 N m^−2^) for the thick foam. These stiffness values were derived from the uniaxial behaviour of the foams using the Hertz contact equation for a cyclindrical indenter and based on the subject's body mass of 77.4 kg. Since OpenSim is restricted to modelling linear behaviour and the polyether polyurethane foam exhibits nonlinear behaviour, an average stiffness value was determined for each foam based on the results of the compression testing. The other contact parameters were set at the following values in each model: dissipation = 0.5 (m s^−1^), static friction = 0.8, dynamic friction = 0.4, viscous friction = 0.4.

In each simulation, the activations of the BFL, RF, VL, VM, TA, LG, MG and SOL MTUs were constrained to match the muscle activities measured experimentally using EMG as much as possible. Residual and reserve actuators were applied to each unlocked degree of freedom in all simulations to provide forces to the model if the MTU actuators were not strong enough to satisfy the externally applied forces. As recommended by Hicks *et al*. [30], we ensured that these reserve actuators provided no more than 5% of the total net moments at each degree of freedom to produce valid simulations of muscle dynamics. The mechanical work generated from each MTU was calculated by integrating the simulated power curves over the entire gait cycle.

## 3. Results

### 3.1. Experimental data

**Figure 2.**
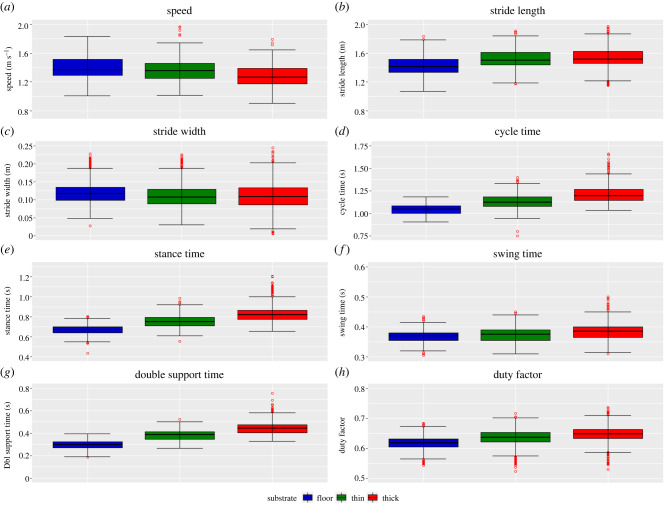
The distribution of spatio-temporal parameters for all participants combined (*n* = 30) while walking on the three different substrates: floor (blue), thin foam (green) and thick foam (red). (*a*) speed, (*b*) stride length, (*c*) stride width, (*d*) cycle time, (*e*) stance time, (*f*) swing time, (*g*) double support time and (*h*) duty factor. Data include all strides for individual trials (*n* = 5023). Red circles denote an individual stride from any subject that represents a statistical outlier.

**Figure 3.**
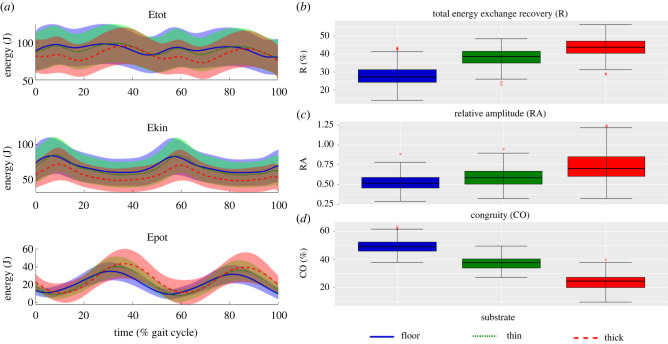
(*a*) Mass-normalized total (*E*_tot_) mechanical energy (top), kinetic (*E*_kin_) energy (middle) and the gravitational potential (*E*_pot_) energy of the COM (bottom) and normalized to walking stride for all participants combined (*n* = 30) while walking on the three different substrates (mean ± s.d) (*n* = 2935). The distribution of pendulum-like determining variables: (*b*) the recovery of total energy exchange as a percentage (R), (*c*) relative amplitude (RA) and (*d*) congruity percentage (CO) for all participants combined (*n* = 30) while walking on the three different substrates. Floor (blue), thin foam (green) and thick foam (red). Red circles denote an individual stride from any subject that represent statistical outlier.

**Figure 4.**
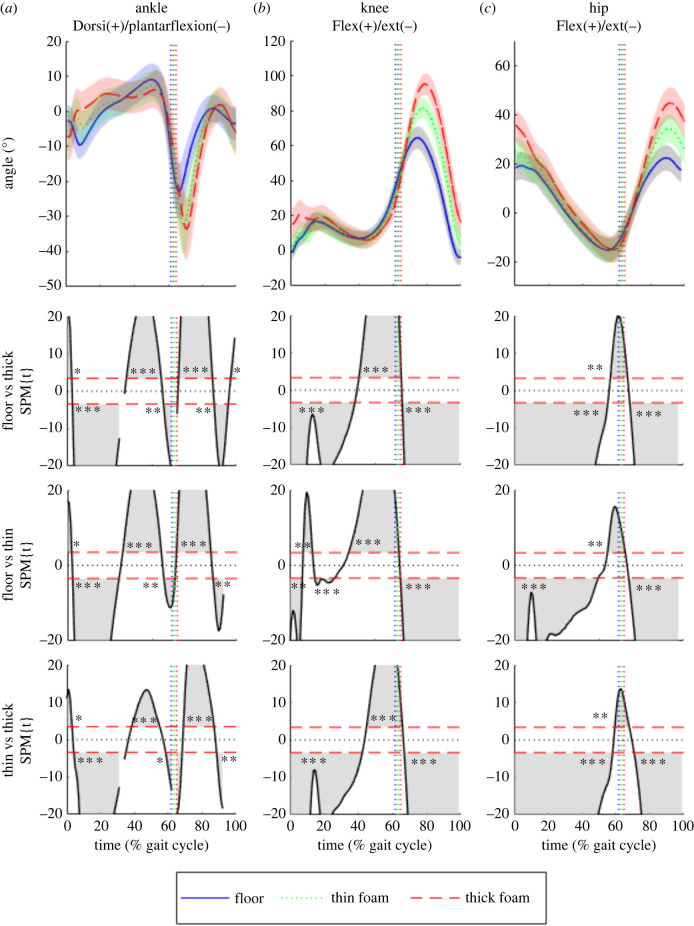
(*a*) Ankle, (*b*) knee and (*c*) hip joint angles in the sagittal plane for all participants combined (*n* = 30) while walking on the three different substrates: floor (blue), thin foam (green) and thick foam (red). The vertical dotted lines indicate toe-off. 1D-SPM (using paired *t*-tests with Bonferroni corrections) indicate regions of statistically significant differences between walking conditions, when 1D-SPM lines exceed the critical threshold values denoted by the horizontal red dotted lines. Shaded regions (within the SPM graphs) correspond to the period within the gait cycle where walking conditions are statistically significantly different from one another.*, **, *** represent *p*-values of less than 0.05, 0.01 and 0.001, respectively.

**Figure 5.**
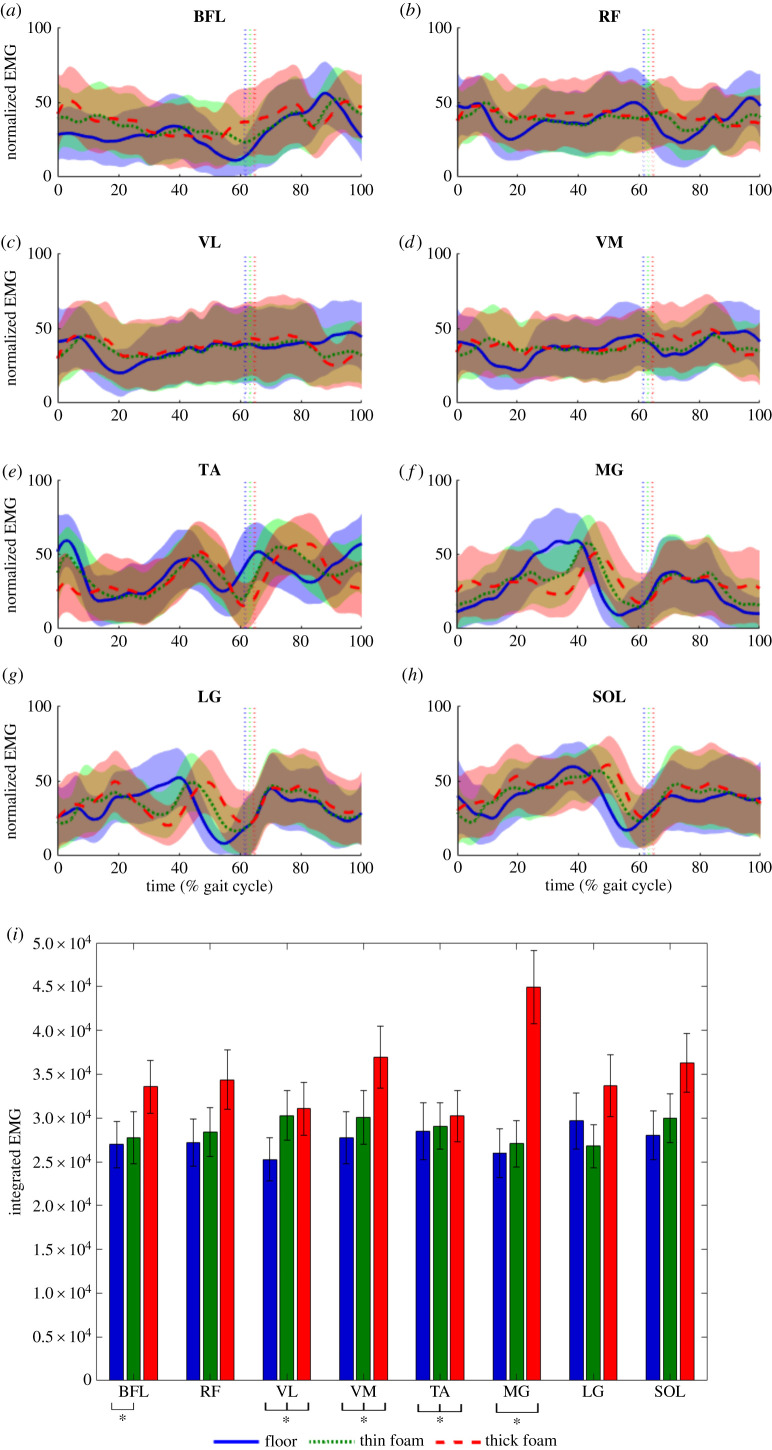
nEMG values for eight left lower extremity muscles for participants combined (*n* = 24) while walking on the three different substrates: floor (blue), thin foam (green) and thick foam (red) (*a*) biceps femoris (BFL), (*b*) rectus femoris (RF), (*c*) vastus lateralis (VL), (*d*) vastus medialis (VM), (*e*) tibialis anterior (TA), (*f*) medial gastrocnemius (MG), (*g*) lateral gastrocnemius (LG) and (*h*) soleus (SOL) (mean ± s.d.). The vertical dotted lines indicate toe-off. (*i*) iEMG values (mean ± s.d.). Asterisks indicates significant differences between substrates (*p* < 0.05).

LMMs found a significant (*p* < 0.001) effect of trial type (continuous walking and single trials) for all spatio-temporal variables (electronic supplementary material, tables S2–S3), joint angles at heel-strike (electronic supplementary material, table S4) and toe-off (electronic supplementary material, table S5) and all iEMG values (electronic supplementary material, tables S6–S7). There were significant (*p* < 0.05) interaction effects between substrate and trial type for most spatio-temporal variables (electronic supplementary material, tables S2-S3), joint angles (electronic supplementary material, tables S4–S5) and iEMG (electronic supplementary material, tables S6–S7). However, for both trial types, substrate effects were similar; therefore, when only individual trial data results are presented visually (figures 2–5), similar differences between substrates also occurred on the continuous trials.

As substrate compliance increased, walking speed and stride width decreased and stride length, cycle time, stance time, swing time and duty factor all increased significantly (*p* < 0.001) (figure 2, electronic supplementary material, tables S2–S3). The coefficient of variation (CV) was similar for speed but decreased by 8% and 12% for stride length between floor and thin and thick foam, respectively. CV increased by 16% and 43% for stride width, 14% and 12% cycle time, 24% and 18% stance time and 28% and 24% swing time between floor and thin and thick foam, respectively (electronic supplementary material, table S8). LMMs found a significant (*p* < 0.001) effect of speed for all spatio-temporal variables and significant (*p* < 0.001) interaction effects between speed and substrate for most spatio-temporal variables (electronic supplementary material, tables S9–S10). LMMs found a significant (*p* < 0.001) effect of gender for stride length and stance time and cycle time, swing time and duty factor (*p* < 0.05). There were significant (*p* < 0.05) interaction effects between gender, speed and substrate for most spatio-temporal variables (electronic supplementary material, tables S9–S10).

When averaged across each subject, *E*_kin_ and *E*_tot_ decreased over most of the stride as substrate compliance increased (figure 3*a*). During most of the stride, *E*_pot_ increased on the foams, except during early to mid-stance (figure 3*a*). As substrate compliance increased, relative amplitude (RA) increased by approximately 4.6% and approximately 33.4% (figure 3*c*) and congruity percentage (CO) decreased by approximately 30% and approximately 18% between floor and thin and thick foams respectively (figure 3*d*). The recovery of the total energy exchange (R) increased by approximately 3.2% between floor and thin foam but decreased by approximately 3.7% between floor and thick foam (figure 3*b*). LMMs showed that the effect of substrate is significant for all variables between most substrates (*p* < 0.05) (electronic supplementary material, table S11). LMMs found a significant effect of speed (*p* < 0.001) and gender for all variables and some significant interaction effects between speed, gender and substrate (*p* < 0.05) (electronic supplementary material, table S11).

1D-SPM analyses of sagittal plane joint angles found significant differences between all substrates at different stages of the stride (figure 4; electronic supplementary material, tables S12–S14). During heel-strike, as substrate compliance increased, there was a significant (*p* < 0.005) increase in hip flexion (figure 4*c*), knee flexion (figure 4*b*) and ankle plantarflexion (figure 4*a*) between all the substrates. LMMs at heel-strike showed that the effect of substrate is significant (*p* < 0.001) for hip angle on all substrates and between floor and thick foam for knee angle (electronic supplementary material, table S15). Also, there was a significant effect of speed for hip angle (*p* < 0.001) and knee angle (*p* < 0.01). At heel-strike, LMMs found no significant (*p* > 0.05) effects for ankle angle (electronic supplementary material, table S15). During early stance, there was significantly less plantarflexion at the ankle joint (*p* < 0.001) on the foams and during late stance, there was less dorsiflexion at the ankle joint (*p* < 0.05) on the foams (figure 4*a*). Throughout much of stance phase, hip and knee joint angles were similar on all substrates. During toe-off, all joint angles were similar but the foot is in contact with the foams for longer. LMMs at toe-off found a significant (*p* < 0.001) effect for knee angle between the floor and thick foam and between floor and thin foam (*p* < 0.05) for ankle and knee angle (electronic supplementary material, table S16). During swing, there were significant increases in plantarflexion at the ankle joint (*p* < 0.01) and in flexion at the knee (*p* < 0.001) and hip joint (*p* < 0.001) as substrate compliance increased (figure 4). There were also some significant (*p* < 0.05) interaction effects between speed, gender and substrate at both heel-strike and toe-off (electronic supplementary material, tables S15–S16).

Overall there was a small increase in muscle activity for all measured muscles as substrate compliance increased (figure 5). However, nEMG for the TA (figure 5*e*) during heel-strike and toe-off and for RF (figure 4*b*), VL (figure 5*c*), VM (figure 5*d*) during heel-strike were higher on the hard floor than on the compliant surfaces. During mid-stance, on the hard floor, nEMG for the MG (figure 5*f*) and LG (figure 5*g*) were also higher than on the foam substrates. This pattern is generally consistent with iEMG values, which show increases for all muscles as substrate compliance increased, except LG on the thin foam (figure 5*i*). LMMs for the iEMG values show the effect of substrate is significant (*p* < 0.01) for VM for all substrates, between floor and thin foam for BFL and LG (*p* < 0.05) and between floor and thick foam for TA (*p* < 0.01) and MG (*p* < 0.001) (electronic supplementary material, tables S17–S18). There was no significant (*p* > 0.05) effect of substrate for RF, VL and SOL. LMMs found a significant (*p* < 0.05) effect of speed for BFL, VL and VM, and gender for BFL, MG and SOL (electronic supplementary material, tables S17–S18). There were also some significant (*p* < 0.05) interaction effects between speed, gender and substrate (electronic supplementary material, tables S17–S18).

### 3.2. Musculoskeletal modelling

**Figure 6.**
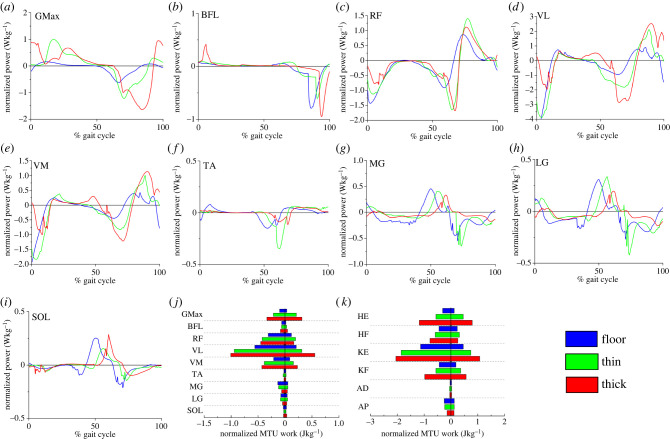
Normalized power (Wkg^−1^; a–i) and mechanical work (Jkg^−1^; *j*) outputs from select lower limb musculotendon units (MTU), as well as functional group totals (*k*), as predicted by subject-specific simulations of walking on the floor as well as the thin and thick foam substrates. Power and work both tended to increase in the more proximal MTUs on the more compliant substrates relative to the floor, however this trend was reversed in the more distal MTUs. GMax- gluteus maximus, BFL- biceps femoris (long head), RF- rectus femoris, VL- vastus lateralis, VM-vastus medialis, TA- tibialis anterior, MG- medial gastrocnemius, LG- lateral gastrocnemius, SOL- soleus, HE- Hip extensors (GMax, BFL, semimembranosus, semitendinosus), HF- Hip flexors (iliacus, psaos, RF), KE- Knee extensors (RF, VL, VM, vastus intermedius), KF- Knee flexors (BFL, biceps femoris short head, semimembranosus, semitendinosus), AD- Ankle dorsiflexors (TA, extensor digitorum longus, extensor hallucis longus), AP- Ankle plantarflexors (MG, LG, SOL, flexor digitorum longus, flexor hallucis longus, tibialis posterior).

The CMC simulations produced valid representations of walking over the hard floor and the foam surfaces. The outputs accurately replicated the energetics of the experimental subject, with estimated CoT values of 2.77, 3.01 and 3.40 J kg^−1^ m^−1^ on the floor, thin and thick foams respectively (compared with experimental values of 2.70, 3.11 and 3.99 J kg^−1^ m^−1^) and a good match between predicted activations and experimental EMG data in the majority of muscles on all substrates (electronic supplementary material, figure S5). Simulations predicted that positive and negative MTU power and work increased with surface compliance in the muscles crossing the hip and knee joints (GMax, BFL, RF, VL, VM; figure 6*a–e*), but decreased in the more distal muscles crossing the ankle (TA, MG, LG, SOL; figure 6*f–i*). Specifically, the peak negative power produced by proximal muscles such as GMax increased from −0.62 W kg^−1^ on the floor to −1.63 W kg^−1^ on the thick foam, while the peak positive power produced by VL increased from 0.89 to 2.51 W kg^−1^ (figure 6*d*). This translated to changes in positive and negative work from 0.03 and −0.10 J kg^−1^ to 0.26 and −0.36 J kg^−1^ on the thick foam in GMax and from 0.20 and −0.55 J kg^−1^ to 0.61 and −0.97 J kg^−1^ in VL (figure 6*j*). These patterns of power and work were different in the distal muscles such as LG, where peak positive power decreased from 0.45 W kg^−1^ on the floor to 0.33 W kg^−1^ on the thick foam (figure 6*h*), which translated to decreases in positive and negative work from 0.04 and −0.07 J kg^−1^ to 0.03 and −0.04 J kg^−1^ (figure 6*j*).

These patterns of power and work in individual muscles were also seen at the functional muscle group level (figure 6*k*). For instance, the hip and knee extensors produced more positive and negative work on the thick foam (hip extensors = 0.57/−0.90 J kg^−1^; knee extensors = 1.18/−2.01 J kg^−1^) relative to the hard floor (hip extensors = 0.12/−0.30 J kg^−1^; hip extensors = 0.46/−1.13 J kg^−1^), while this pattern was reversed in the ankle plantarflexors (thick foam = 0.11/−0.13 J kg^−1^; floor = 0.12/−0.25 J kg^−1^).

## 4. Discussion

It has long been recognized that animals incur a higher energetic cost when moving on compliant substrates like sand, snow and foam [6–8,10,13]. However, as noted by Davies & Mackinnon [10], the methods and data used to elucidate the underlying mechanical causes of this increase have varied considerably in the literature, while substrate properties are rarely quantified. By collecting a comprehensive and relatively large experimental motion dataset we were able to systematically characterize changes in walking gait with substrate compliance, and, by combining data with mechanical substrate testing, drive the first subject-specific computer simulations of human locomotion on compliant substrates to estimate the altered internal kinetic demands on the musculoskeletal system. These analyses lead us to reject a number of previous hypotheses related to increased locomotor costs, and instead lead us to modify other previous mechanisms to propose a more intricate explanatory model for increased energetic costs of walking on compliant terrains.

Our LMMs show that gender and walking speed have significant interaction effects in our statistical models of spatio-temporal parameters and energy exchange variables (electronic supplementary material, tables S9–S11). However, we find no significant difference in CoT between males and females on any substrate (electronic supplementary material, figure S6), which is consistent with previous findings on hard substrates [31]. Furthermore, in a previous study, we found no statistically significant relationships between CoT and various morphological variables that are likely to have gender biases such as lower limb length, body stature and maximum isometric ankle plantarflexion torques [16]. Given these results, and more importantly that the qualitative differences in kinematics between substrates are the same for males and females, we conclude that gender does not influence this examination of the causative mechanisms underpinning CoT increases on the foams generally and universally across the cohort. Walking speed has an instrinsic mechanistic link with most gait parameters and as such it is not suprising that significant interaction effects are recovered in the LMMs. Average walking speeds were 1.36, 1.32 and 1.23 m s^−1^ on the floor, thin and thick foams respectively, and these differences are recovered as statistically significant. However, studies of changes in CoT with walking speeds on hard substrates recover small increases in CoT as speed increases across the range observed here (e.g. [32]), in contrast to our negative relationship between CoT and speed. Given this different polarity of change in CoT, and the small magnitude of speed change, we suggest that as an isolated variable, speed is not a important causative contributor to the observed increase in CoT across the substrates.

Walking is most efficient when the whole-body CoM moves in an inverted pendulum motion, allowing for an optimal exchange of kinetic and potential energy between gait cycles [20]. It has been proposed (HYP1) that disruptions to the inverted pendulum mechanics of walking contribute to the observed increase in energetic costs on compliant substrates such as sand [6]. However, in this study we observed little differences in the recovery of total energy exchange (R) with 57–61% R found across all substrates (figure 3). Lejeune *et al*. [7] also found a relatively efficient pendular mechanism when walking on sand with as much as 60% mechanical energy recovery despite sand having low resilience. Our findings suggest that there is little to no disruption to the inverted pendulum mechanics of walking on compliant substrates. We therefore reject HYP1.

The mechanical work needed to move CoM is directly related to the cost of walking, particularly at step-to-step transitions [33,34]. Stance phase is important as it requires active braking with the absorption of external power, followed by active propulsion to allow the CoM to be directed toward the opposite side. Pontzer *et al*. [35] found a strong correlation between CoT and estimated volume of muscle activated per metre travelled. Based on previous work, we hypothesized that increased muscle activation either (HYP2a) throughout the limb [3] or (HYP2b) within specific muscle groups [14] was responsible for increased energetic costs on compliant terrains. Overall we saw increased activation in all measured muscles (figure 5), partially supporting HYP2a. Bates *et al*. [14] previously suggested that walking on compliant substrates will increase energetic costs as greater muscle-tendon forces are required by the ankle extensors to generate the propulsion needed from mid-stance to reaccelerate into the swing phase. In partial support of this, we found slightly increased ankle extensor values during terminal stance or push-off on the foams. However, our computer simulations suggest there is no increase in the mechanical work done by the TA (figure 6*f*), MG (figure 6*g*), LG (figure 6*h*) and SOL (figure 6*i*) during mid-stance to push-off on these compliant substrates compared with the hard floor. These findings (and others; see below) indicate that, while muscle activations do increase on compliant terrains, these increases do not uniformly or simplistically translate into increased locomotor costs, suggesting HYP2 is too simplistic as a standalone explanation.

In similar vein, we find partial support for (HYP3) increased MTU work and decreased efficiency, but our results (figure 6) emphasize a much more complex pattern across MTUs on compliant substrates [7,8]. While our simulations predicted that positive and negative MTU power and work increased with substrate compliance in muscles crossing the hip and knee joints (GMax, BFL, RF, VL, VM; figure 6*a–e*), a decrease (contra HYP3) was predicted in the more distal muscles crossing the ankle (figure 6). These patterns of muscle activation (figure 5) and power production (figure 6) are related to the significant kinematic differences on the three substrates, most notably at heel-strike and during swing (figures 2–4). When the joints are more flexed and less aligned with the resultant ground reaction force, a greater volume of active muscle is required [35]. In particular, increased hip and knee flexion is clearly mechanistically related to greater mechanical work done by the muscles crossing the knee and hip joints (Gmax, BFL, RF, VL, VM) (figure 6). Previous studies have suggested that walking on uneven or irregular terrain [1,3,4] also incurs increased mechanical work at the knee and hip due to greater knee and hip flexion, and thus the patterns of muscle activation and force production recovered here may apply to other terrain types with elevated energetic costs.

The nature and magnitude of changes in ankle joint kinematics are consistent with the little or no increase in mechanical work seen in distal limb muscles in our simulations (figure 6). Here, a larger total joint excursion (i.e. the range of motion through both greater maximum dorsiflexion and plantarflexion angles) is observed on the hard floor during stance rather than foams, where ankle angle remains relatively constant during midstance (figure 4*a*) compared with the continuous dorsiflexion observed on the hard floor. nEMG data (figure 5*a*) suggests greater activation of LG, MG and to a lesser extent SOL during midstance on the hard floor, with active dorsiflexion of the ankle suggesting that activation of these muscles is eccentric versus near-isometric on the foams (figure 4*a*). As a result, these muscles are predicted to incur greater negative mechanical power and work during stance on the hard floor compared with the foams (figure 6). Therefore we propose that previous hypotheses that changes in muscle kinetics and energetics (HYPs 2 and 3; [3,7]) should be refined, and that increased mechanical work at the knee and hip due to greater flexion and overall joint excursion is primarily responsible for increased energetics costs on compliant substrates, with negligible contribution from distal muscles.

These changes to joint kinematic and associated muscle kinetics are mechanistically related to the changes observed in spatio-temporal gait parameters (figure 2). We found that more compliant substrates resulted in significant increases in stride length, cycle time, stance time, swing time and duty factor, but decreases in speed and stride width (figure 2). Cotes & Meade [36] found an increase in step length resulting in greater vertical displacements of the CoM. Previous simulation [37] and experimental [33] studies also concluded that larger steps increased energetic costs due to CoM redirection. Slower stride frequencies, rather than reduced stride length, account for the observed slower speeds. However, previous studies on slippery surfaces have observed slower walking speeds with shorter stride lengths and flatter foot-floor angles at heel-strike, possibly to keep the CoM centred over the supporting limb to improve stability [38,39]. The increase in cycle time, stance time, swing time and duty factor are partly due to the reduction in speed; however, the increase in duty factor on compliant substrates suggests there is a proportionally longer stance time. As peak ground reaction forces will be lower on compliant substrates, an increase in stance time ensures there is enough time to exert force on the ground to redirect the CoM. This reduction in efficiency for the redirection of the CoM would produce an increase in mechanical work and thus, consume more metabolic energy. Similar mechanisms are observed in smaller animals [40], in young children [41] and adults walking on uneven terrain [3,4] who adopt a more crouched gait, coupled with an increase in stance time, to ameliorate the power costs. These changes are ultimately inter-linked with the postural or kinematic changes (figure 4), and their muscular mechanisms (figure 6) observed here (see below).

It was also hypothesized that (HYP4) correcting greater instabilities indicated by increased variability in gait [13] increase energetic costs. While there was no change in CV for speed and a decrease in CV for stride length, we found large increases in CV for stride width, cycle time, stance time and swing time on the compliant foams compared with the hard floor (electronic supplementary material, table S8). However, while previous studies have correlated increased step-to-step variability with increased CoT, they have noted that even relatively high levels of variability yield modest increases in metabolic costs [42,43]. For example, O'Connor [42] found that a 65% increase in step width variability was correlated with a 5.9% increase in energetic costs. Here we find lesser increases in CV for stride width on the foam but greater increases in CoT. Therefore, while we find support for HYP4, we infer that changes in hip and knee joint kinematics and kinetics represent the major contributor to increased CoT on compliant substrates.

Here, we chose foams as the focus substrate and through material testing of mechanical properties we were able to simulate locomotion on compliant terrain using a highly detailed musculoskeletal model for the first time. This leads us to present an explanatory model of CoT increase in which elevated activity and mechanical work by muscles crossing the hip and knee are required to support the changes in joint (greater excursion and maximum flexion) and spatio-temporal kinematics (longer stride lengths, stride times and stance times and duty factors) on compliant substrates. Other compliant substrates, such as sand (and indeed even other types of foams) probably exhibit different mechanical properties to our foams, in addition to other responses (e.g. foot slippage [6]) and therefore the extent to which our explanatory factors apply universally to compliant terrains remains to be tested. Huang *et al*. [44] found that reduced ankle push-off, and greater collisional losses, resulted in greater positive work throughout the gait cycle, as well as compensations at the other joints, particularly at the knee joint. Furthermore, they found increased mechanical work at the lower limb joints resulted in greater energy expenditure, in support of our proposed model [44]. We hypothesize that the modified joint kinematics and spatio-temporal kinematics, and associated increase in muscle work at the hip and knee, are likely to occur (albeit to varying degrees) on most compliant substrates in healthy adult subjects, and therefore the model of CoT increase we present here will be widely applicable for similar human populations, and potentially mammals more widely where relatively upright limb postures are used. It would also be interesting for future work to explore changes in musculoskeletal mechanics on compliant substrates in animals that utilize more crouched postures. For example, birds typically use considerably less hip motion than humans and power the stride predominantly from the knee and ankle joints [45]. It is therefore possible that greater responses to changes in substrate compliance may be observed in distal, rather than proximal, joints in birds and other animals with crouched postures.

## 5. Conclusion

Our analyses lead us to reject a number of previous hypotheses related to increased locomotor costs, such as disruptions to the inverted pendulum mechanics and increased mechanical work at distal limb muscles. Instead we find that the mechanistic causes of increased energetic costs on compliant substrates lie predominantly in the proximal limb and are more complex than captured by any single previous hypothesis. Specifically, elevated activity and greater mechanical work by muscles crossing hip and knee are required to support the changes in joint (greater excursion and maximum flexion) and spatio-temporal kinematics (longer stride lengths, stride times, stance times, duty factors and increased variability) on our compliant substrates. The validation of a computer simulation of locomotion on compliant substrates herein demonstrates the potential of this approach to explore morphological and mechanical adaptations to different substrates in other animal groups.

## Data Availability

Experimental data and code for analysis and figure generation are available from the Dryad Digital Repository: https://doi.org/10.5061/dryad.6hdr7sr31 [46]. Data are available in the electronic supplementary material [47].
